# Two Patients with Colic

**Published:** 1894-09

**Authors:** Chas. G. Wilson

**Affiliations:** Clarksville, Tenn.


### TWO PATIENTS WITH COLIC.

Chas. G. Wilson, M. D., Clarksville, Tenn.

Last spring I had two patients with colic or neuralgia in abdomen and stomach. Both cases were women, slender, dark complexion, vivacious. The colic usually came on two or three hours after a meal. Less during winter than summer. Diet did not affect it, but unloosing the corset gave temporary relief. Symptoms of both were about same. Hot flashes, with smothering and cold perspiration on forehead. Dislike for pork, but no bad effect from eating it. Late going asleep and late awakening. Then has bad taste in mouth. Used to like sour things but not now. Late scanty menses with some cramping at times. Both cases had no more attacks after a dose of Puls.$^{200}$ although there had been no complete cessation in two years in one and three years in the other. There had been much drugging done, with finally a dose of Antikamnia when most severe. I relate these cases in hope they may aid some one in selecting the right remedy.

